# Time-Dependent, HIV-Tat-Induced Perturbation of Human Neurons *In Vitro*: Towards a Model for the Molecular Pathology of HIV-Associated Neurocognitive Disorders

**DOI:** 10.3389/fnmol.2017.00163

**Published:** 2017-05-29

**Authors:** Kim T. Gurwitz, Richard J. Burman, Brandon D. Murugan, Shaun Garnett, Tariq Ganief, Nelson C. Soares, Joseph V. Raimondo, Jonathan M. Blackburn

**Affiliations:** ^1^Division of Chemical and Biological Systems, Department of Integrative Biomedical Sciences, University of Cape TownCape Town, South Africa; ^2^Division of Physiological Sciences, Department of Human Biology, University of Cape TownCape Town, South Africa; ^3^Neurosciences Institute, University of Cape TownCape Town, South Africa; ^4^Institute of Infectious Disease and Molecular Medicine, University of Cape TownCape Town, South Africa

**Keywords:** neuronal cell culture, HIV-Tat, mass spectrometry, proteomics, whole-cell patch clamp, intrinsic neuronal properties, HIV-associated neurocognitive disorders

## Abstract

A significant proportion of human immunodeficiency virus type 1 (HIV)-positive individuals are affected by the cognitive, motor and behavioral dysfunction that characterizes HIV-associated neurocognitive disorders (HAND). While the molecular etiology of HAND remains largely uncharacterized, HIV transactivator of transcription (HIV-Tat) is thought to be an important etiological cause. Here we have used mass spectrometry (MS)-based discovery proteomics to identify the quantitative, cell-wide changes that occur when non-transformed, differentiated human neurons are treated with HIV-Tat over time. We identified over 4000 protein groups (false discovery rate <0.01) in this system with 131, 118 and 45 protein groups differentially expressed at 6, 24 and 48 h post treatment, respectively. Alterations in the expression of proteins involved in gene expression and cytoskeletal maintenance were particularly evident. In tandem with proteomic evidence of cytoskeletal dysregulation we observed HIV-Tat induced functional alterations, including a reduction of neuronal intrinsic excitability as assessed by patch-clamp electrophysiology. Our findings may be relevant for understanding *in vivo* molecular mechanisms in HAND.

## Introduction

The introduction of combination antiretroviral therapy (CART) in 1996 has greatly reduced human immunodeficiency virus-1 (HIV)-related mortality and has improved quality of life for HIV-positive individuals. However, HIV associated co-morbidities persist in the CART era and present a challenge for those treating or living with HIV infection (Brew, 2004). One group of complications that affects 18%–60% of HIV-positive individuals, are the HIV-associated neurocognitive disorders (HAND; Cysique and Brew, 2011; Joska et al., 2011a,b). This is a group of disorders resulting from HIV infection of the central nervous system (CNS), characterized by varying degrees of cognitive, motor and behavioral dysfunction (Antinori et al., 2007).

While CART has mediated a shift toward less severe forms of HAND, the prevalence of HAND remains high due to a number of factors, including the increased longevity afforded by CART, incomplete penetrance of antiretroviral drugs into the CNS (allowing for long-term viral replication and subsequent neurotoxicity) and potential antiretroviral-mediated neurotoxicity (Brew, 2004; Valcour et al., 2004; Letendre et al., 2008; Ances et al., 2010; Heaton et al., 2010; Simioni et al., 2010). Furthermore, the more subtle symptoms that characterize less severe forms of HAND are still clinically significant (Antinori et al., 2007).

At present, no rapid reliable diagnosis exists for this HIV co-morbidity and the exact molecular pathology—required for biomarker identification and rational drug design—is largely unknown. While the true etiology of HAND is likely complex (González-Scarano and Martín-García, 2005), HIV transactivator of transcription (HIV-Tat) protein appears particularly significant (King et al., 2006). An improved understanding of the proteomic effects of the HIV-Tat protein on human neurons may shed light on this phenomenon.

HIV-Tat is HIV’s transcription factor and is a 86–100 residue protein comprising two exons; the first contains all known functional regions of the protein and is highly conserved (72 amino acid residues) and the second is of variable length (depending on the HIV subtype) and unknown function (Tahirov et al., 2010). *In vitro* cell culture experiments have shown that HIV-Tat is capable of inducing apoptosis in mammalian cells (Eugenin et al., 2003), but the biological relevance of this to HAND is uncertain since synaptodendritic injury has been shown to be a better clinical correlate of HAND than frank neuronal loss (Masliah et al., 1997) and is, moreover, believed to be a reversible process (Everall et al., 1999; Bellizzi et al., 2005; Ellis et al., 2007). Further, HIV-Tat exposure has been linked to impaired learning and memory—processes known to involve synaptic dynamics (Goellner and Aberle, 2012)—as well as gray matter deficits in mice (Carey et al., 2012, 2013). These observations point to HIV-Tat’s direct involvement in the HAND phenotype and suggest a functional consequence of HIV-Tat exposure on neurons. Elucidation of the molecular effects of HIV-Tat on neurons is therefore important for a better understanding of HAND pathogenesis, as well as for identifying novel, plausible, damage-reversal strategies.

It is well known that HIV-Tat is able to penetrate the cell membrane (Frankel and Pabo, 1988; Ma and Nath, 1997) and directly modulate host transcription and translation, resulting in global changes in host-cell function. For example: a genome-wide chromatin immunoprecipitation sequencing (ChIP-seq) experiment revealed that HIV-Tat extensively binds the host genome of T lymphocytes (Marban et al., 2011); and specific host factors have exhibited altered expression as a result of HIV-Tat treatment *in vitro* (Flores et al., 1993; Buonaguro et al., 1994). While other HIV proteins, such as HIV-Vpr, can negatively affect neuronal physiology, HIV-Tat seems particularly important in the pathogenesis of HAND as it is the only HIV protein actively secreted by infected primary immune cells in the CNS (Nath, 2002; Perry et al., 2010; Na et al., 2011).

The aging HIV-positive population—as a result of the increased longevity afforded by CART (Brew, 2004) and the stabilizing infection rate (UNAIDS, 2013)—incentivizes the standardization of diagnosis and prognosis of HAND (for example, by identifying predictive markers of disease progression; de Jager et al., 2015), as well as the identification of plausible drug targets in order to develop novel long-term HAND therapeutic strategies. Here, we aimed to contribute to this effort by characterizing the effect of HIV-Tat treatment on non-transformed human neuronal cells *in vitro* over time. We hypothesize that it is the cumulative effect of HIV-Tat exposure to many neurons that may contribute to the development of HAND. The first 48 h of infection of individual cells might reveal clues for how to treat or prevent HAND progression. If one looks at later time points, it might be too late as the cell may have already apoptosed, the damage of which is irreversible. Here, we employed mass spectrometry (MS)-based proteomics to quantify differentially expressed protein groups between treated and control samples over time (6, 24, and 48 h, respectively) and have identified HIV-Tat-induced proteomic changes reflecting cytoskeletal dysregulation and changes to gene expression machinery. In addition, we have demonstrated a functional implication of these proteomic changes by observing HIV-Tat induced reduction in intrinsic neuronal excitability.

## Materials and Methods

### Cell Culture

The cell line used in this work is a non-transformed, neuroepithelial-like stem (NES) cell line derived from 5-week-old (Carnegie stage 15–17) human fetal hindbrain, generously donated by Prof Austin Smith’s group at the Cambridge Stem Cell Institute, UK. The NES cells were maintained and differentiated as described in Tailor et al. (2013). Briefly, cells were seeded at approximately 26,000 cells/cm^2^ on non-pyrogenic, cell adhesion plates coated with poly-L-ornithine (0.01% w/v in phosphate buffered saline (PBS), Sigma Aldrich) and laminin (0.2% v/v in PBS, Sigma Aldrich) and incubated at 37°C, 95% humidity, and 5% carbon dioxide (CO_2_). The NES cells were cultured in DMEM:Hams F12 medium (Sigma Aldrich) supplemented with L-glutamine (2 mM, Sigma Aldrich), penicillin/streptomycin (1% v/v, Lonza), N2 (1:100 v/v, Invitrogen), B27 (1:1000 v/v, Invitrogen), and epidermal growth factor (EGF) and fibroblast growth factor 2 (FGF2; both 0.01 ng/μl, Invitrogen; Tailor et al., 2013). The growth medium was changed daily. For cell culture passage of cells in the stem cell state, cells were lifted with tryp-LE™ (Invitrogen) and split 1:3 once a density of approximately 70,000 cells/cm^2^ was reached (~90% cell culture confluence, determined visually), which occurred roughly every third day. The cells were differentiated at ~90% cell culture confluence unless otherwise specified.

For differentiation, NES cells were grown until 90% cell confluence was reached, at which point EGF and FGF2-containing growth medium was removed and replaced with growth medium lacking growth factors and containing a higher concentration of B27 (1:100 v/v), which induced spontaneous NES differentiation (Tailor et al., 2013). The differentiation medium was identical to the stem cell culture medium except for the above-mentioned changes. Cells were washed with PBS before addition of differentiation medium to ensure removal of growth factors. Cells were incubated at 37°C, 95% humidity, and 5% CO_2_. During the differentiation process and during maintenance of differentiated neuronal cultures, the medium was changed twice weekly. All cell culture experiments were performed in triplicate for each experimental condition.

### Immunohistochemistry

For immunohistochemistry experiments, a single T-75 flask (Sigma CLS3290) of proliferating NES cells was split to fill all wells of two 24-well cell adhesion dishes (Sigma CLS3527). Cells were then allowed to proliferate in neuronal stem cell medium for 24 h in order to settle. Each plate was differentiated as described above and labeled for immunohistochemistry assays on days 2, 4, 8, or 12 post differentiation. During labeling, the cell culture medium was removed from each of the wells and the cells were washed with PBS. Cells were then fixed with 4% paraformaldehyde for 10 min and again washed with PBS. The wells were blocked for 1 h with blocking solution (0.3% Triton-X-100 and 5% goat serum in PBS) at room temperature followed by another PBS wash. The fixed cells were incubated with primary antibodies (as per experimental condition) in 0.1% triton and 1% bovine serum albumin (BSA) for 12 h. Primary antibodies used were mouse IgG2A anti-β-III-Tubulin (anti-TuJ-1; R&D Systems, MAB1195, 1:200)—a neuronal marker—and sheep anti-Glial fibrillary acidic protein (GFAP; R&D Systems, AF2594, 1:50)—a glial marker. Wells were then washed three times with PBS followed by a 1-h incubation with fluorophore conjugated secondary antibodies, as well as DAPI. Secondary antibodies (Alexa fluorophores from Invitrogen) were as follows: goat anti-mouse IgG2A conjugated to green Alexa Fluor 488 for anti-TuJ-1, and donkey anti-sheep IgG conjugated to red Alexa Flour 555 for anti-GFAP. DAPI was used to stain cell nuclei “blue”, for all experimental conditions.

### Cell Imaging

The NES cells were visualized using phase contrast microscopy (20× objective) on a Zeiss Axiovert 200M Fluorescence microscope with a Zeiss AxioCam high-resolution monochrome camera and images were captured using Zeiss AxioVision software (version 4.8). Immunohistochemistry images were captured on a LEIKA inverted fluorescent microscope (Leica DMI 4000), at ×20–63 magnification, and processed with the Leica Application Suite Advanced Fluorescence software.

### HIV-Tat Treatment and Harvesting of Treated Cells

For HIV-Tat treatments, differentiated NES cells, which had been plated on 10 cm cell adhesion dishes (Sigma CLS430167), were treated on day 9 post differentiation. Differentiated NES were treated with a final concentration of 1 ng/μl clade B (86 amino acid) HIV-Tat (Diatheva), suspended in a final concentration of 0.1 mM beta mercaptoethanol (BME; vehicle concentration optimized previously; Ganief et al., 2017). NES cells treated with 0.1 mM BME only, were used as a control. Replica differentiated neuronal cultures (*N* = 3 for each experimental condition) were incubated with the treatment/control at 37°C, 95% humidity, 5% CO_2_ and were harvested after 6, 24 or 48 h. After incubation, as per experimental time point, cells were lifted using tryp-LE™ (Gibco), washed with PBS and pelleted at 600 g. Supernatants were aspirated and cell pellets flash frozen in liquid nitrogen.

### Sample Preparation for MS Analysis

Cell pellets were lysed in the presence of 1× protease inhibitors (Sigma Aldrich) and 1× phosphatase inhibitors (PhosStop, Roche) using a 1× Tris/HCl pH 7.6 radioimmunoprecipitation assay (RIPA) buffer (Holden and Horton, 2009) excluding 10 mM dithiothreitol (DTT), which otherwise interferes with bicinchoninic acid (BCA) protein quantitation. Cell lysates were incubated at 4°C for 1 h with 1 μl Benzonase nuclease ultrapure (Sigma Aldrich). Subsequently, lysates were centrifuged at 14,000 *g* for 10 min and supernatants containing the cell protein content were transferred to new 1.5 ml plastic tubes. Protein quantitation was then performed using the Pierce™ BCA Protein Assay Kit microplate procedure (ThermoScientific) with BSA as the standard.

Tryptic peptides for MS analysis were generated on 30 kDa molecular weight cutoff (MWCO) filters (Millipore) using the Filter-Aided Sample Preparation (FASP) protocol (Wiśniewski et al., 2009). Briefly, 30 μg protein—as determined by BCA quantitation—was added to each filter. Samples were incubated with trypsin (1:50 w/w, Promega) for 12 h and resultant peptides were eluted in accordance with the FASP protocol and subsequently acidified with 0.1% formic acid (FA) in ultrapure dH_2_O (both Sigma Aldrich). Samples were desalted using reverse-phase C18 chromatography (C18 columns manufactured in-house from C18 discs, Millipore) prior to MS analysis. Briefly, C18 columns were equilibrated with 80% MS grade acetonitrile (ACN, Sigma Aldrich) acidified with 0.1% FA. Ten microgram peptide from each sample was loaded on separate C18 columns—one column per sample—and desalted by the addition of 2% ACN acidified with 0.1% FA. Finally, peptides were eluted in to glass vials with 60% ACN acidified with 0.1% FA. Desalted peptides were dried at room temperature using a SpeedyVac (Savant) and resuspended in 0.1% FA (Sigma Aldrich) in ultrapure dH_2_O to a final concentration of 250 ng/μl, which was estimated from BCA quantitation at the protein level on the assumption of no protein loss during FASP.

### LC-MS2 Analysis Parameters

Liquid chromatography tandem mass spectrometry (LC-MS2) analysis was performed on a Q Exactive MS instrument coupled to a Dionex Ultimate 3500 RSLCnano ultra high-pressure nano LC system (both Thermo Fischer Scientific). All samples were injected into the sample loop at a flow rate of 5 μl/min in 2% buffer B (0.1% FA/ACN) and 98% buffer A (0.1% FA/ultrapure dH_2_O). Samples were then loaded onto a 40 cm, 75 μm diameter, C18 reverse phase analytical column (3.6 μm diameter Aeris Peptide C18 packing material, packed in house; Phenomenex 04A-4507) and subsequently eluted during a 120-min segmented gradient (4%–30% buffer B) at a flow rate of 400 nl/min. At the end of each sample run, buffer B was ramped to 80% for 10 min, which was then followed by a 20-min wash cycle, from 2% to 50%, and a 20-min column equilibration. After every fourth sample, a longer wash (80 min) was run. Mass spectra were collected with settings adapted from Nel et al. (2015) with the following amendments: intensity threshold for ion selection was set at 6.3e3; isolation window for MS2 was set at 2.0 m/z; and MS2 spectra were acquired at a target value of 5e4.

### Whole-Cell Patch-Clamp Electrophysiology

At 9 days post-differentiation, NES cells were treated with HIV-Tat or vehicle for 48 h (as above). The cells, attached to cell adhesion dishes, were removed from the incubator and transported rapidly to the recording chamber of a Zeiss Axioskop Upright Microscope (Zeiss) for electrophysiological recordings. Recordings were made in NES cell differentiation medium (as described above and in Tailor et al., 2013) at room temperature. All recordings were obtained during the first 2 h following NES cell removal from the incubator environment. A horizontal puller (Model P-1000, Sutter) was used to pull patch pipettes (13–20 MΩ tip resistance) from filamental borosilicate glass capillaries (2.00 mm outer diameter, 1.58 mm inner diameter, Hilgenberg, Germany). The pipettes were filled with an internal solution comprising (in mM): K-gluconate (126); KCl (4); Na 2 ATP (4); NaGTP (0.3); Na 2-phosphocreatinine (10) and HEPES (10). Osmolarity was adjusted to between 290 mOsM and 300 mOsM and the pH was adjusted to between 7.38 and 7.42 with KOH. NES cells were visualized with a 40× water-immersion objective (Zeiss) and digital images were obtained with a charge couple device (CCD) camera (VX55, TILL Photonics). Recordings were made in current clamp and voltage clamp mode with an Axopatch 200B amplifier (Molecular Devices). Data acquisition was performed through an ITC-1600 board (Instrutech) connected to a personal computer running custom-written clamp sequences (PulseQ) under IGOR Pro (Wavemetrics). Analysis was performed using custom-written MATLAB (Mathworks) scripts.

### Data Analysis

Raw mass spectra were processed for peptide peak identification and quantitation by MaxQuant software (version 1.5.03; Cox et al., 2009). Default MaxQuant settings were used with the following modifications: labeling was set to singlet, label free quantitation (LFQ) was selected for relative LFQ, “match between runs” was selected, and only peptides unique to a protein group were used for quantitation. MaxQuant’s inbuilt search engine, Andromeda (Cox et al., 2011), was used for protein group identification using the UniProt human proteome database as the reference (FASTA, downloaded on 12/01/15, containing 121996 sequence entries). The MS proteomics data have been deposited to the ProteomeXchange Consortium^1^, via the PRIDE partner repository (Vizcaíno et al., 2014), with the dataset identifiers PXD006314 and PXD006332; the data is freely available.

The data generated by MaxQuant were filtered to exclude protein groups that mapped to a sequence in the decoy database or that mapped to a known contaminant. In addition, only protein groups that had an LFQ measurement in each experimental sample, and had a satisfactory posterior error probability (PEP) score (<0.01), were taken forward for downstream data analyses.

MS data quality and summary statistics were assessed with the aid of a script developed by Karsten Krug (Proteome Center Tübingen, University of Tübingen, Germany) in the R environment for statistical computing^2^. Using the LFQ data from the triplicate biological repeats of treated and control samples, differential expression analysis was performed by hypothesis testing, using the independent two-sampled *t*-test (*p* < 0.05) for each protein group mean LFQ value (HIV-Tat treated vs. control) to identify differentially expressed protein groups.

### Biological Significance Analysis

Functional classification analysis and gene annotation were performed using *Panther*^3^ (Thomas et al., 2003; Mi et al., 2013a,b), and *GeneCards*^4^, respectively. Gene names, corresponding to differentially expressed protein groups (from the comparison of HIV-Tat treated vs. control conditions) were used for the analyses. *Panther* protein class was used to classify protein groups into functional groupings in order to reveal broad molecular pathways affected by HIV-Tat treatment over time. In *Panther* Gene List Analysis, the following parameters were selected: (1) “ID list” selected as “list type”; (2) Species set to *Homo sapiens*; and (3) functional classification viewed on bar chart. The *Panther* interface allows one to view bar charts of *Panther* protein class ontologies at progressively lower *Panther* protein class terms, thereby corresponding to more and more specific groupings. Bar charts were recreated, at various hierarchical levels, as line graphs in R (using scripts developed in-house) in order to visualize the change in number of genes that map to a particular *Panther* protein class classification over time. Each bar chart, per time point—in which each bar mapped to a particular *Panther* protein class—was also viewed as a table of genes (from the input list of differentially expressed protein groups). *GeneCards* was then consulted to functionally annotate the genes belonging to each grouping. Gene function was correlated to log_2_ fold change (HIV-Tat/control) to gain insight as to the effect of HIV-Tat treatment on the neuronal proteome.

Further, statistical over-representation analysis (*Panther* version 10.0, released 15/05/15) of *Panther* protein classes per time point was carried out to determine significantly over/under represented Panther protein classes (and their corresponding molecular pathways) when compared to the background. The following parameters were user-specified: organism set to H. sapiens; list of differentially expressed genes, per time point, was used as the “analyzed list”; full list of genes identified, per time point, was used at the “reference list” (i.e., background); and Bonferroni correction was set to “false”.

## Results

### Neuroepithelial-Like Stem Cells Differentiate into Functional Neurons

**Figure 1:**
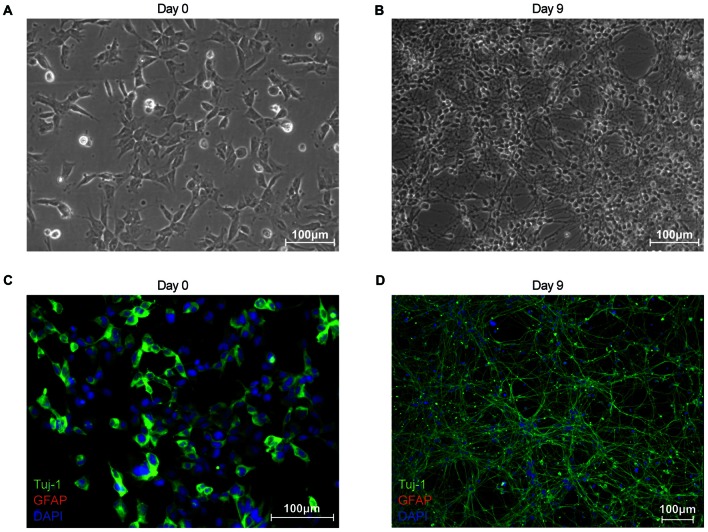
Characterization of proliferating and differentiated neuroepithelial-like stem (NES) cells in terms of morphology and cell-type marker expression. **(A)** Phase contrast image of proliferating NES (day 0). **(B)** Phase contrast image of differentiated NES (day 9 post differentiation). **(C)** Proliferating NES cells (day 0) stained for: β-III-Tubulin (TuJ-1; green Alexa Fluor 488); glial fibrillary acidic protein (GFAP; red Alexa Flour 555); and nuclear DNA (blue DAPI stain). **(D)** Differentiated NES cells (day 9 post differentiation) stained for: TuJ-1 (green Alexa Fluor 488); GFAP (red Alexa Flour 555); and nuclear DNA (blue DAPI stain).

**Figure 2:**
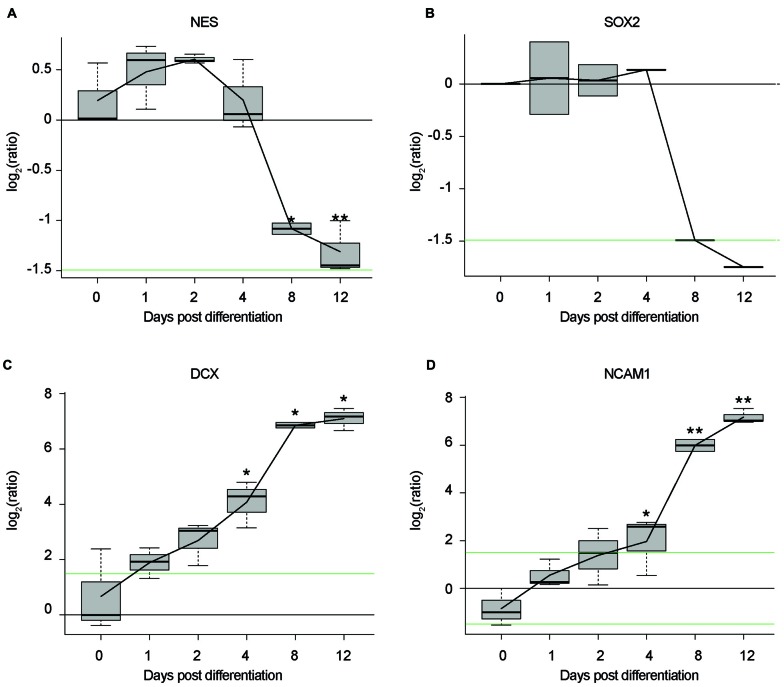
Time-course, proteomic data of NES cells during differentiation. Log_2_ ratios of average protein expression at each time point, divided by average protein expression at Day 0. **(A)** Expression of Nestin (NES) over time. **(B)** Expression of Sox2 over time. **(C)** Expression of double cortin (DCX) over time. **(D)** Expression of neural cell adhesion molecule 1 (NCAM1) over time. All replicates in triplicate. *p < 0.05. **p < 0.01. *P* values were generated by a two-sided *t*-test between the three replicates from Day 0 and the three replicates from each of the differentiation day time points. Black line: log_2_ = 0. Green lines: log_2_ = 1.5|−1.5.

In this study, we establish differentiated NES cells as a cell culture model for studying the molecular effects of HIV-Tat on human neurons. It is evident that NES cells grow as a monolayer and form characteristic rosette-like groupings in their stem cell state (Figure 1A), as described previously by Tailor et al. (2013). Further, in accordance with the original characterization of this cell line (Tailor et al., 2013), the cells spontaneously differentiate when growth factors EGF and FGF2, are removed from the growth medium (once the desired cell culture confluence is reached—typically 90% confluence). After 9 days of differentiation there are drastic changes to cell morphology, such as decreased cell body size and extensive neuronal network formation (Figure 1B). These cells do not express GFAP—a known glial specific marker (Gorris et al., 2015)—in either their proliferating (Figure 1C) or differentiated state (Figure 1D), indicating that these cells differentiate into neurons and not a mixed neuron/glial population. Assertions that these cells differentiate into non-proliferative neurons, were further corroborated by proteomic data, which showed (1) decrease in expression of known neuronal stem cell markers (Gorris et al., 2015) over time—for example, Nestin (Figure 2A) and Sox2 (Figure 2B)—and (2) increase in expression of known neuronal commitment markers (Gorris et al., 2015)—for example, Double Cortin (DCX; Figure 2C) and Neural Cell Adhesion Molecule 1 (NCAM1; Figure 2D) (Garnett and Blackburn, unpublished). In addition, changes in protein expression had begun to taper off between days 8 and 12 post differentiation (Figure 2; Garnett and Blackburn, unpublished), indicating that the differentiation changes had begun to stabilize at the chosen time point for treatment (i.e., 9 days post differentiation). Therefore, in accordance with what has been previously described for this cells line (Sun et al., 2008; Tailor et al., 2013), the NES cells closely respond to growth cues from the medium, supporting the assumption that they are not genetically immortalized. Whole-cell patch-clamp analysis demonstrated the presence of spiking activity in over 70% of recorded cells confirming the neuronal phenotype of these cultures. Differentiated NES therefore represent a realistic, well-characterized cell culture model of neurons in the human brain.

### Mass Spectrometry Reveals Widespread Proteomic Changes in Response to HIV-Tat Treatment

We first set out to perform an unbiased analysis of HIV-Tat induced proteomic changes using quantitative MS-based methods. Differentiated NES cells (day 9) at 90% cell culture confluence were treated with exogenous HIV-Tat and harvested at 6, 24, and 48 h post-treatment. HIV-Tat treatment concentration, as well as treatment and harvesting time points, were selected based on literature precedent and previous work in our laboratory (Xiao et al., 2000; Nath, 2002; Ganief et al., 2017). Of the 1 067 211 MS2 spectra submitted to Max Quant software for analysis, 568 656 (53.28%) were mapped to known tryptic peptide sequences in the Uniprot Human protein database corresponding to an identification of 4104 protein groups in total, across the 20 samples analyzed (false discovery rate <0.01), and a minimum of 2319 protein groups identified in each sample (for protein group data, see Supplementary Information 1 (S-1)). Considering a recent estimate that 3000 proteins constitute ~99% of the protein mass of a mammalian cell (Wiśniewski et al., 2012), it was encouraging to observe 2319 protein groups common to all samples. The low percentage of contaminants (0.41% of total protein groups), the low percentage of matches to the decoy database (0.038% of total protein groups), and good tryptic digestion (i.e., few missed cleavages, as seen in Supplementary Information 2 (S-2), Figure S-2.1) were evidence of the high quality of collected data. Additionally, average absolute mass error was relatively low and consistent across samples suggesting technical stability (Supplementary Information 2 (S-2) Figure S2.2). This proteomic data is accessible in the PRIDE database.

**Figure 3:**
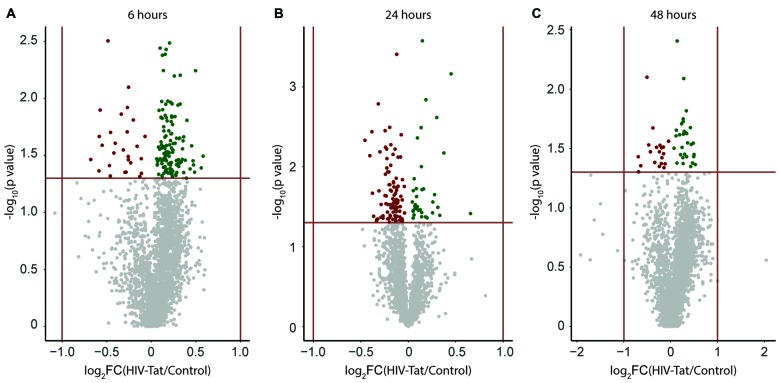
Visual representation of expression changes between human immunodeficiency virus type 1 transactivator of transcription (HIV-Tat) treated and Control (vehicle only treated) samples. Protein groups are plotted as a function of significance (−log_10_ *p* value) and log_2_ fold change (log_2_FC). **(A)** 6 h post treatment **(B)** 24 h post treatment **(C)** 48 h post treatment. FC: fold change = HIV-Tat/Control. Horizontal red line indicates *p* = 0.05 (level of significance set for the independent *t*-test analysis per protein group). Vertical red lines indicate two-fold change in expression (±log_2_1). Up-regulated protein groups are colored “green”, down-regulated protein groups are colored “red”.

A large number of protein groups were expressed differentially between HIV-Tat and control samples at each time point post treatment. This differential expression can be represented as a volcano plot (Figure 3). Upon visual inspection of the volcano plots, it is apparent that the magnitude of differential expression is subtle (Figure 3, −1 < log_2_ fold change <1), but statistically significant (*p* < 0.05) for many protein groups. A total of 131, 118, and 45 protein groups were identified (Supplementary Information 1 (S-1)) as being significantly differently expressed at 6, 24, and 48 h post HIV-Tat treatment, respectively. These differentially expressed protein groups were then further analyzed to determine the temporal changes in protein expression in response to HIV-Tat treatment, as well as the potential biological significance of such changes, since they relate to HIV-Tat’s effect on neurons in cell culture in our *in vitro* model of HIV infection of the CNS.

### HIV-Tat Treatment Results in the Dysregulation of Cytoskeletal Maintenance Proteins

**Figure 4:**
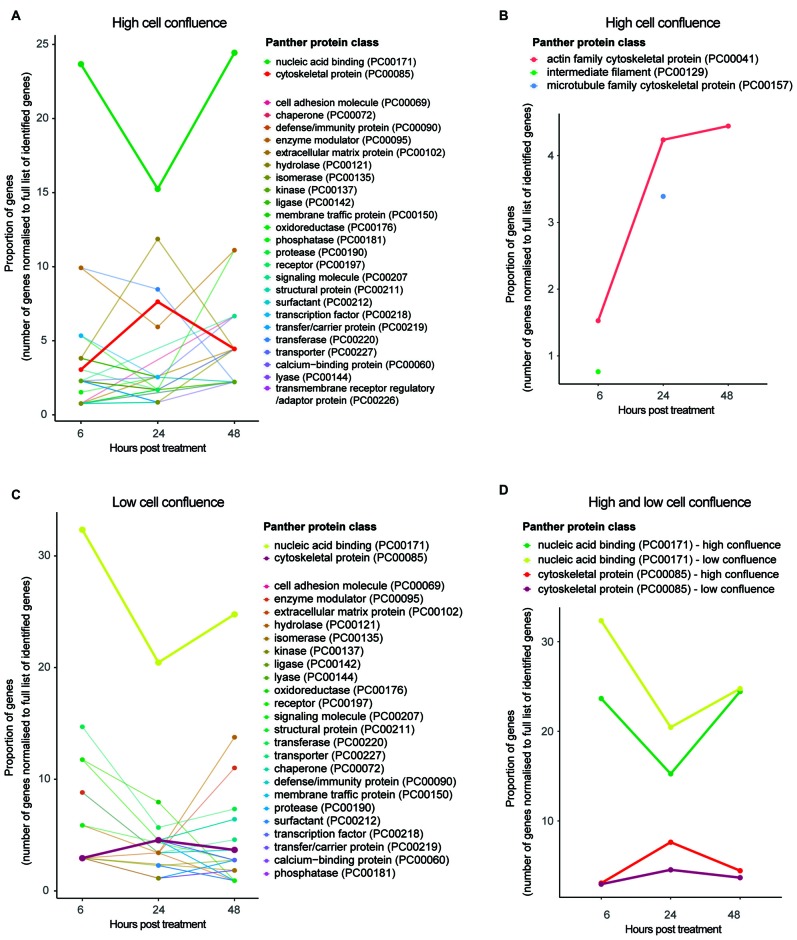
Functional classification of protein groups differentially expressed due to HIV-Tat treatment over time. **(A)** High cell culture confluence experiment. **(B)** *Cytoskeletal protein* subclasses for high cell culture confluence experiment. Please note, proteins mapping to *intermediate filament* (PC000129) and *microtubule* protein (PC000157) protein subclasses were not observed at every time point. **(C)** Low cell culture confluence experiment. **(D)** Trends of differentially expressed protein groups that map to the protein classes *nucleic acid binding* and *cytoskeletal protein* across time and cell confluence (high vs. low). High confluence: cells differentiated at 90% confluence. Low confluence: cells differentiated at 70% confluence. y axis: number of genes normalized to the number of differentially expressed genes per time point. Gene name used as proxy for protein group. Protein classes refer to *Panther* protein classes (http://www.pantherdb.org/).

Functional classification of protein groups significantly differentially expressed between time points—in accordance with *Panther* protein class^5^—revealed those biological functions affected by HIV-Tat treatment over time (Figure 4A). Of particular interest in this analysis are protein groups that map to the *Panther* protein class *cytoskeletal protein* (PC00085). While more protein groups map to this classification at 24 h post treatment than at 6 or 48 h post treatment (from inspection of protein groups belonging to *cytoskeletal protein* subclasses), the proportion of dysregulated *actin related* proteins in fact increases over time (Figure 4B). Rather than representing cytoskeletal building block components (e.g., actin, microtubules, intermediary filaments), most protein groups that map to this protein class are cytoskeletal maintenance/cytoskeleton anchoring proteins (e.g., *CTNA1* at 6 and 48 h, which links the cytoplasmic domain of membrane cadherins to actin at adherens junctions; GeneCards^6^; Supplementary Tables S-2.1, S-2.3) or cytoskeletal transport proteins (e.g., *KLC2* and *ARP10* at 24 h; Supplementary Table S-2.2).

The observation that proportionally and progressively more actin related proteins are differentially expressed over time (Figure 4B) is in agreement with continued repression of these protein groups (negative log_2_ fold change expression values at all three time points; Supplementary Tables S-2.1–2.3) and suggests that the HIV-Tat-dependent effect on cytoskeletal instability is sustained, perhaps strengthening over time. This observation is supported by over/under representation analysis, in which the protein class *cytoskeletal protein* is significantly under represented at 6 h post treatment (*p* = 5.14e-2) and the protein class *extracellular matrix protein* (PO00102)—involved in synaptic plasticity and physically linked to the actin cytoskeleton via cell adhesion molecules (Geiger et al., 2001)—is significantly over represented at 48 h post treatment (*p* = 2.26e-2; Supplementary Tables S-2.10–2.12). It is worth clarifying that one may observe down-regulation (in terms of log_2_ fold change) of a set of protein groups, but over representation of the protein class—to which the set of protein groups belong—in comparison to the background proteome (i.e., Fischer’s exact test). This scenario would indicate that the implicated protein class (or broader function) is targeted by the treatment specifically and that the HIV-Tat treatment down-regulates many of the individual members of this protein class.

### Cell Density Affects HIV-Tat Mediated Cytoskeletal Dysregulation in NES Cells

The abovementioned time course experiment (cultured cells differentiated at 90% cell culture confluence and then treated at 9 days post differentiation) was repeated with cells differentiated at lower cell culture confluence (70%) in order to determine the effect of cell density on HIV-Tat treatment (for protein groups information, see Supplementary Information 1 S-1, including Figures S-1.1–1.4). While trends across time were comparable across the two different cell culture confluences in terms of the proportion of protein groups mapping to the protein class *cytoskeletal protein* (Figure 4D), cells differentiated at 70% confluence showed no significant effects on the cytoskeleton upon HIV-Tat treatment (Supplementary Tables S-2.13–2.15). Whilst, it appeared that an effect to the cytoskeleton did occur, this was not strong enough to meet threshold requirements for statistically significant over-representation (in comparison to background) at lower cell densities.

### HIV-Tat Affects Differentiated Neurons at the Level of Gene Expression Machinery

Next, we examined the effect of HIV-Tat on gene expression machinery. When the *Panther* protein class *nucleic acid binding* (PC00171) was considered for both high (90%) and low (70%) cell culture confluence experiments, we observed proportionally fewer differentially expressed protein groups that map to this classification at 24 h post treatment, than at 6 and 48 h post treatment (Figure 4D). In addition, most protein groups in the *nucleic acid binding* list were down-regulated at 24 h after HIV-Tat treatment (i.e., negative log_2_ fold change), while the few that were up-regulated in response to treatment are involved in mRNA turnover and RNA degradation (Supplementary Table S-2.5). Complementary patterns are seen in the data from the 70% cell confluence experiment (Supplementary Table S-2.17). Taken together, these trends suggest general repression of gene expression by HIV-Tat at 24 h post treatment. This is in contrast to what was observed at the 6 h post treatment time point, where differentially expressed protein groups that map to this protein class were mostly up-regulated (i.e., positive log_2_ fold change) in response to HIV-Tat treatment (Supplementary Tables S-2.4, S-2.16).

To give specific examples from the high cell culture confluence experiment, these up-regulated protein groups are involved in chromatin remodeling (e.g., *SMRC2*) and DNA replication (e.g., *MCM5* and *RFA1*) at the DNA level, as well as RNA splicing (e.g., *PRP4*), mRNA processing (e.g., *GMPPA*) and translational machinery (e.g., *RL10*, *RL21*, *RL5*, *RS2*) at the RNA level. This suggests an up-regulation of general host gene expression by HIV-Tat at 6 h post treatment. Most protein groups in this class were also up-regulated at 48 h post treatment (Supplementary Table S-2.6), which according to their putative biological function again suggests a general increase in host gene expression at this time. Complementary patterns, are seen in the data from the 70% cell confluence experiment (Supplementary Table S-2.18). It seems reasonable to suppose that changes to *nucleic acid binding* upon HIV-Tat treatment would not be affected by cell confluence, as this class of protein groups is largely involved with *intracellular* processes. Cytoskeletal and synaptic maintenance however, may very well be affected by the number of cells present and consequent levels of communication between cells, as described above.

Since there are more RNA and DNA binding proteins than all other proteins in mammalian cells, including brain cells (Gerstberger et al., 2014), one might expect that proteins belonging to the *nucleic acid binding* class would always end up having the largest proportion of protein groups mapped to it (in comparison to other protein classes (Figures 4A,C)). Importantly, we found that the protein class *nucleic acid binding* itself was not significantly over or under represented when compared to background (Supplementary Tables S-2.10–2.15). However, significantly over or under represented protein classes that are functionally linked, and share the same parent lineage to this broad class, were apparent. For example, the class *RNA methyltransferase* (PO00033) was over represented 5-fold at 6 h post treatment for the higher cell culture confluence experiment (*p* = 5.52e-3, Supplementary Table S-2.10), the class *DNA binding protein* (PO00009) was under represented less than 0.2-fold at 24 h post treatment (*p* = 2.47e-2, Supplementary Table S- 2.11), and the class *DNA binding protein* (PO00009) was over represented 2.34-fold at 48 h post treatment (*p* = 6.08e-2, Supplementary Table S-2.12). Similar over/under representation patterns were seen for the lower cell culture confluence experiment (Supplementary Tables S-2.13–2.15).

In addition, our over/under representation data correlated well with expression change trends (log_2_ fold change). That is to say, an over representation of a protein class functionally linked to *nucleic acid binding* was associated with up-regulation of the specific protein groups that map to *nucleic acid binding* (Supplementary Tables S-2.4–2.6, S-2.10–2.12). The combined weight of these observations makes it unlikely that the size of this category biased the observed trend in *nucleic acid binding* protein groups in response to HIV-Tat treatment. In summary, we observed a consistent U-shaped response in the expression of *nucleic acid binding* proteins. An initial up-regulation at 6 h post HIV-Tat treatment was followed by down-regulation at 24 h, before up-regulation again at 48 h.

### Individual Protein Groups Were Differentially Expressed at Multiple Time Points Following Application of HIV-Tat

In order to explore potential pathways relevant to HIV-Tat’s effect on neurons, we identified several protein groups which were differentially expressed at more than one time point. Of interest were those that were up-regulated at a single time point, then down-regulated at a second time point—and that correlated with implicated broader biological function classifications. For example, *MRPL45* (involved in mitochondrial protein translation) and *DDX18* (an RNA helicase) are both up-regulated at 6 h post HIV-Tat treatment and subsequently become down-regulated at 24 h post treatment (Supplementary Information 2, Table S-2.7). These protein groups both map to the *Panther* protein class *nucleic acid binding*, which as explained above exhibits a U-shaped response in expression over time after HIV-Tat treatment. The observation that *many* individual protein groups followed this trend in expression over time further strengthens the hypothesis that HIV-Tat treatment causes an initial up-regulation and subsequent down-regulation of gene expression at 6 and 24 h post treatment, respectively. Although not of particular interest to the themes addressed in this manuscript, a handful of significantly differentially expressed proteins were also observed at 24 and 48 h post treatment (Supplementary Table S-2.8).

In addition, certain protein groups were differentially expressed at both 6 *and* 48 h post treatment (*CO3A1*, *CTNA1*, *SNX27*; Supplementary Table S-2.9). As these protein groups were all present in the “total list” of all protein groups “seen” by the MS instrument at the 24-h time point (see Supplementary Information 1 S-1), this phenomenon cannot be explained by these protein groups simply not being detected by the MS instrument at 24 h post treatment. Rather they were not *significantly* differentially expressed between treated and control conditions at 24 h post treatment. It is therefore noteworthy that these proteins were significantly differentially expressed at 6 h and then again at 48 h post HIV-Tat treatment, implying that this was not a prolonged effect over 48 h, but that the same gene expression changes occurred *again* at a second time point. This recurrence of the effect is supported by the observation that the same “direction” of expression regulation (positive or negative log_2_ fold change) occured at the second time point. The genes that encode these protein groups are not located on the same chromosome (*GeneCards*), so the trend cannot be explained by co-expression due to proximity of genetic loci. It is interesting to note that all protein groups differentially expressed at both 6 and 48 h post HIV-Tat treatment were cytoskeletal or membrane-associated proteins (Supplementary Table S-2.9).

### HIV-Tat Significantly Affects Functional Properties of Neurons 48-h After Treatment

**Figure 5:**
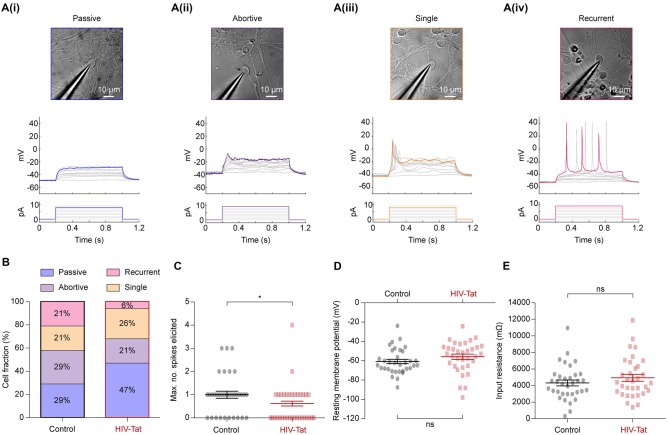
HIV-Tat treatment reduces the intrinsic excitability of NES cells. **(A)** Whole-cell patch-clamp recordings were performed on a total of 68 cells (34 in each group) following either 48 h of treatment with HIV-Tat or vehicle (“Control”). Cells were targeted for recording using differential interference contrast imaging (top). Recordings were conducted in current-clamp mode and spiking patterns (middle) identified following step-wise injection of current (bottom). Elicited firing patterns could be divided into four categories that included **(i)** “passive”, **(ii)** “abortive”, **(iii)** “single” and **(iv)** “recurrent” spiking patterns. We did not observe any correlation between cell morphology and recorded firing pattern in the cells selected. **(B)** The fraction of cells in each of the defined firing pattern categories. Note that HIV-Tat treatment resulted in fewer cells in the more excitable categories. **(C)** There was a significant decrease in the maximum number of spikes elicited from the HIV-Tat treated cells compared to the control cells. There was no significant difference in resting membrane potential **(D)** nor in the input resistance **(E)** between the control and treatment groups. Error bars denoted mean ± *SEM*, *p = 0.02, *Mann-Whitney U-test*.

Finally, we sought to determine whether HIV-Tat induced changes in protein expression correlated with any functional changes to neurons in the form of alterations to neuronal excitability or other intrinsic properties. To do so, we performed whole-cell patch-clamp recordings from cells 48 h after either HIV-Tat or vehicle treatment (named “Control”). Patch-clamp recordings for both groups were performed in standard media and not in the presence of HIV-Tat. Cells were targeted for recordings based on their morphology. Cells with a small round or ovoid shaped cell body (5–10 μm) with two or more extended processes were chosen (Figure 5A).

In current-clamp mode, current steps of between 0 pA and 10 pA were applied. Action potentials (or spikes) representing the “output” of a neuron are an important measure of neuronal function and equate to the intrinsic excitability of a neuron. Individual cells showed different types of spiking responses which could be separated into four categories: a purely passive (Figure 5A(i)), abortive spike (Figure 5A(ii)), single spike (Figure 5A(iii)) and recurrent spiking response (Figure 5A(iv)). These spiking response properties are similar to those seen in other hESC-derived neurons (Moore et al., 2009), iPSC-derived neurons, and acute human fetal brain slices (Perrier et al., 2004; Vazin et al., 2009; Belinsky et al., 2011).

Post-mitotic neurons mature and regulate their function by inserting appropriate ion channels into the plasma membrane, particularly voltage-gated ion channels (Moody and Bosma, 2005). Therefore, the observed spiking response to current injection can be used to determine the excitability of a neuron: passive (least excitable) → abortive spike → single spike → recurrent spikes (most excitable). Spiking responses were collected from 34 control and 34 HIV-Tat treated cells. Although cells from all four categories were observed in both conditions, HIV-Tat treatment resulted in a larger fraction of cells within functionally “less excitable” categories (Figure 5B). For example, HIV-Tat treatment resulted in a significant increase in the number of cells in the “passive” category and decrease cells in the “recurrent spikes” category as compared to control (passive 29% vs. 47%, recurrent 21% vs. 6%; *p* = 0.00013, *X*^2^ *test*). There was no significant difference noted between the “abortive spikes” and “single spikes” between the two groups (abortive 29% vs. 21%, single 21% vs. 26%; *p* = 0.19, *X*^2^ *test*). A related finding was that, the maximum number of spikes that could be elicited following current injection was significantly smaller (Figure 5C: 0.59 ± *SEM* 0.1408 spikes vs. 1.029 ± *SEM* 0.16 spikes) in the HIV-Tat as compared to control condition (*p* = 0.021, *Mann-Whitney U-test*). Interestingly, there was no significant difference in resting membrane potential (Figure 5D: −60.77 ± *SEM* 2.24 mV vs. −55.88 ± *SEM* 2.73 mV; *p* = 0.17, *unpaired t-test*) nor in the input resistance between the control and HIV-Tat treated cells (Figure 5E: 4318 ± *SEM* 358.2 Ω vs. 4944 ± *SEM* 422.7 Ω; *p* = 0.26, *unpaired t-test*). This suggests that neurons treated with HIV-Tat were equally viable as control neurons given that a loss of a membrane potential is a sign of ensuing cell death. It is important to note that recordings were not performed in the presence (or absence) of HIV-Tat in the recording media which is known to acutely depolarize neurons (Cheng et al., 1998; Brailoiu et al., 2008).

**Figure 6:**
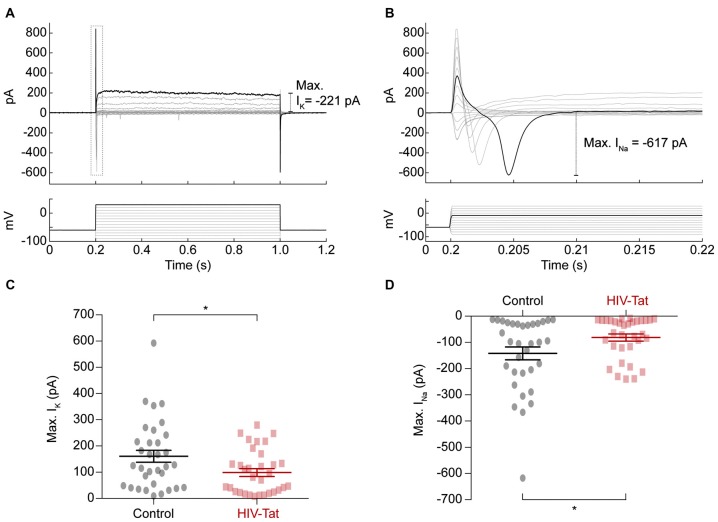
HIV-Tat treatment reduces the magnitude of elicited sodium and potassium currents. **(A)** Whole-cell patch-clamp recordings in voltage-clamp mode were performed and membrane currents (top) were recorded following 10 mV voltage steps between −90 mV and 30 mV (bottom). Dashed gray box demonstrates how voltage-gated sodium currents were measured (enlarged in “**B**”). **(C)** HIV-Tat treated cells were found to have significantly reduced potassium currents (I_K_) compared to control. **(D)** HIV-Tat caused a significant reduction in the magnitude of voltage-gated sodium currents (I_Na_). Error bars denote mean ± *SEM*, *p < 0.05, *Mann-Whitney U-test*.

To explore the underlying changes to ion channel function, which resulted in HIV-Tat induced changes in functional excitability we performed voltage-clamp recordings (Figure 6) to assess the size of elicited sodium (Na^+^) and potassium (K^+^) currents respectively (Figures 6A,B). These recordings showed that the Na^+^ currents (Figure 6C) were significantly smaller in the HIV-Tat treated cells compared to the control cells (-79.96 ± 24.84 pA vs. −145.2 ± 13.58 pA; *p* = 0.035, *Mann-Whitney U-test*). Similarly, the K^+^ currents (Figure 6D) were also significantly smaller in the HIV-Tat treated cells (93.76 ± 13.64 pA vs. −131.7 ± 22.93 pA; *p* = 0.02, *Mann-Whitney U-test*). This suggests that HIV-Tat reduces the neuronal excitability by down-regulating ion channel expression, particularly voltage-gated sodium channels.

## Discussion

We have used unbiased, quantitative MS-based proteomics to identify previously unknown, cell-wide changes in protein expression induced by HIV-Tat in a non-transformed, human neural stem cell model of HAND, our goal being *inter alia* to shed new light on the plausible mechanistic role of HIV-Tat in HAND. We have made our extensive proteomic data available on the PRIDE database; this now represents a useful resource for those interested in the molecular pathology of HAND. Between 45 and 131 protein groups were identified as being significantly differently expressed at various time points following HIV-Tat treatment. We noticed obvious dysregulation of proteins involved in cytoskeletal maintenance and gene expression. These changes were correlated with disruption of neuronal function in the form of reduced intrinsic excitability.

Our study design aims to model the direct interaction of HIV-Tat on neurons over relatively short timescales (<2 days) and does not attempt to recapitulate other important elements of HAND, such as chronic inflammatory responses from other cell types such as microglia and astrocytes (King et al., 2006). This simplifies the interpretation of proteomic data where all HIV-Tat induced changes to protein expression can be attributed exclusively to neurons. Alterations in the expression of cytoskeletal maintenance proteins represents a group of potentially relevant proteomic changes we observed in our dataset. The cell cytoskeleton underlies multiple definitive features of neurons including dendrites and the distribution of ion channels at specific locations such as the axon initial segment or postsynaptic density (Lai and Jan, 2006). As a result, the cytoskeleton is an important determinant of neuronal function including intrinsic neuronal excitability and synaptic activity dynamics (Lin and Koleske, 2010). The dysregulation of cytoskeletal maintenance proteins we observed upon HIV-Tat treatment, particularly under conditions of high cell density and enhanced synaptic connectivity, would be expected to have important functional implications. Data from previous *in vitro* studies using HIV-Tat support this line of reasoning. For example, HIV-Tat has been shown to shorten neurite outgrowths (Rahimian and He, 2016) and cause loss of synapses (Kim et al., 2008). Compromise of the neuronal cytoskeleton and consequent synaptodendritic injury is thought to be a more reliable indicator of cognitive impairment of HIV-infected individuals as opposed to frank neuronal loss (Masliah et al., 1992, 1997; Moore et al., 2006; Ragin et al., 2012). Indeed, many other neurological conditions, such as mental retardation and spastic paraplegias, have been linked to cytoskeletal damage (Goellner and Aberle, 2012). It is therefore plausible that something similar may occur during the molecular pathogenesis of HAND. As synaptodendritic damage in HAND has been shown to be reversible (Ellis et al., 2007; Ragin et al., 2012), it may be beneficial to focus on cytoskeletal dysregulation as a target for prophylactic drug development or early biomarker identification to address symptoms prior to the occurrence of irreversible injury.

A second major finding of this work was HIV-Tat induced alterations in expression of nucleic acid binding proteins. We hypothesize that the pattern of observed changes may be explained by competition between HIV-Tat and the host. Initially (as measured at 6 h post treatment), HIV-Tat up-regulates expression of protein groups with *nucleic acid binding* activity directly, through its transcription factor activity, or indirectly, by activation of other proteins, such as host transcription factors. This is plausible, given HIV-Tat’s role as a transactivator of transcription for the HIV genome and its ability to bind many human-genome loci and to recruit and bind many host factors (Flores et al., 1993; Buonaguro et al., 1994; Gatignol, 2007; Marban et al., 2011; Debaisieux et al., 2012). *Nucleic acid binding proteins* are presumably targeted by HIV-Tat, as these proteins comprise the transcriptional and translational machinery of the cell, much of which is recruited by HIV-Tat for HIV genome replication. This initial up-regulation of gene expression machinery protein groups is followed by an overall down-regulation of protein groups—as well as fewer proteins differentially expressed—at 24 h post treatment. This, to extend the hypothesis, perhaps indicates a host response attempting to curb HIV-Tat’s effects and regain host cell homeostasis. Then, as HIV-Tat degrades in the cell as per its half-life and/or as it is actively cleared (both of which are not well documented in the literature), gene expression machinery may increase again to compensate for previous decreased functioning. One could further hypothesize that gene expression machinery proteins will no longer be differentially expressed, and will reach baseline levels, at time points beyond 48 h, assuming no additional active HIV-Tat is added to the system. Alternatively, the up-regulation of gene expression machinery at 48 h post treatment may be a second, slower response that is perhaps only apparent after 48 h. This points to the importance of extending the time course shown in the current work to understand longer-term effects of HIV-Tat and cell recovery post-exposure. If this second proposed response does continue long-term, one might draw parallels between the trends in nucleic acid binding proteins in HIV-Tat treated neurons and other biphasic genomic responses in nature, such as the *Mycobacterium tuberculosis* (Mtb) hypoxic response (Rustad et al., 2008). Importantly, it is known that in such biphasic genomic responses to external stimuli, there can be little overlap in the genes involved in the two waves of the response (Rustad et al., 2008), perhaps supporting the notion of a similar biphasic response mechanism occurring within the gene expression machinery of HIV-Tat treated neurons.

Finally, our electrophysiological recordings at 48 h post HIV-Tat treatment demonstrate a functional effect of the multiple protein expression changes elucidated using MS. We observed a reduction in the intrinsic excitability of neurons exposed to HIV-Tat driven by a reduction in voltage-gated sodium currents. Unfortunately, membrane-bound and membrane-associated proteins (such as voltage-gated sodium channels) are difficult to detect using MS. This is due to their association with lipids, which impede their isolation and solubilization in buffers suitable for MS (Jahn et al., 1989). Nonetheless, it is highly likely that the HIV-Tat induced changes in cytoskeletal regulation contribute to the reduction in intrinsic excitability we observed. This is because voltage-gated ion channels are trafficked and targeted to the neuronal membrane by the cytoskeleton itself. Previous work has demonstrated that HIV-Tat promotes network excitability by directly depolarizing neurons (Cheng et al., 1998; Brailoiu et al., 2008; Zucchini et al., 2013) via activation of the glutamatergic NMDA receptor. Ca^2+^ influx via activated NMDA receptors is thought to be a common mechanism underlying both synaptodendritic injury and neurotoxicity (King et al., 2006). Interestingly, HIV-Tat induced loss of synapses is thought to represent an adaptive response to increased excitability within neural networks (Kim et al., 2008). In some cases, cell cultures have been shown to adapt to prolonged exposure of HIV-Tat in order to maintain consistent levels of excitation despite the depolarizing effects of the peptide (Krogh et al., 2014). Our observation of a reduction in intrinsic excitability and spiking propensity may constitute a mechanism underlying this effect, whilst also reflecting an obvious functional perturbation of neuronal activity, which may be relevant to cognitive disturbances in HAND.

Taken together, our data reveal novel molecular mechanisms with potential relevance for HAND. We have provided a proteomic resource of HIV-Tat induced molecular perturbations which are independent of brain inflammation. We have demonstrated a dysregulation of cytoskeletal maintenance proteins, which is likely to manifest as cytoskeleton instability. This is supported by electrophysiological measurements which indicate a functional consequence of HIV-Tat exposure on the intrinsic excitability of neurons. We also provide proteomic evidence indicating that HIV-Tat has a complex effect on the gene expression machinery of the neuronal host. Further, protein groups differentially expressed in response to HIV-Tat treatment at multiple time points may represent potentially important molecular switches and are good targets for follow up validation work in the identification of molecular biomarkers for the characterization or treatment of HAND. The functionally relevant neuronal cell culture model of HAND, established in this work, has important implications for HAND research in the diagnosis and treatment of this common HIV co-morbidity.

## Author Contributions

KTG: HIV-Tat cell culture experimental work and mass spectrometry proteomic experimental work, analysis of data, writing of manuscript; RJB: electrophysiology experimental work, data analysis thereof, write up of electrophysiology sections; BDM: cell culture experimental work for electrophysiology experiments and writing of the manuscript; SG: immunohistochemistry experimental work, cell culture and mass spectrometry experimental work and analysis for neuronal differentiation experiments, assistance with cell culture and proteomic experimental work and data analysis for HIV-Tat experiments; TG: assistance with cell culture and proteomic experimental work, data analysis and biological significance analysis for HIV-Tat experiments; NCS: technical supervision and assistance; JVR: technical supervision, data analysis and writing of the manuscript; JMB: corresponding author, technical supervision, data analysis and writing of the manuscript.

## Conflict of Interest Statement

The authors declare that the research was conducted in the absence of any commercial or financial relationships that could be construed as a potential conflict of interest.

## Footnotes

^1^http://proteomecentral.proteomexchange.org

^2^http://www.r-project.org/

^3^www.panther.org

^4^www.genecards.org

^5^www.pantherdb.org

^6^www.genecards.org

## Supplementary Information

### Supplementary Information 1 (S-1)

Summary data and MaxQuant outputs for protein groups identified in both high and low cell confluence experiments.

### Supplementary Information 2 (S-2)

For cell culture experiment at 90% confluence:

Supplementary Figure S-2.1 shows percentage missed cleavages for the higher cell culture confluence experiment. Supplementary Figure S-2.2 shows average absolute mass error of the MS instrument per run for the higher cell culture confluence experiment.

Classifications of differentially expressed protein groups per time point—according to Panther protein class—can be viewed in Supplementary Tables S-2.1–S-2.3 (*cytoskeletal protein* PC00085) and Supplementary Tables S-2.4–S-2.6 (*nucleic acid binding* proteins PC000171). Protein groups differentially expressed at more that one time point can be viewed in Supplementary Tables S-2.7–S-2.9. Over/under representation analysis outputs per time point can be observed in Supplementary Tables S2.10–S-2.12.

For cell culture experiment at 70% confluence:

Over/under representation analysis outputs per time point can be observed in Supplementary Tables S-2.13–S-2.15. Classifications of differentially expressed protein groups per time point—according to Panther protein class—can be viewed in Supplementary Tables S-2.16–S-2.18 (*nucleic acid binding* proteins PC000171).

## Abbreviations

BME: beta mercaptoethanol (control)
CART: combination antiretroviral therapy
CNS: central nervous system
FA: formic acid
FASP: filter aided sample preparation
GFAP: glial fibrillary acidic protein
HAND: HIV associated neurocognitive disorders
HIV: human immunodeficiency virus type 1
HIV-Tat: HIV transactivator of transcription
LFQ: label free quantitation
MS: mass spectrometry
MS2: tandem mass spectrometry
MWCO: molecular weight cutoff
NES: neuroepithelial like stem cells
PEP: posterior error of probability
TuJ-1: β-III-Tubulin.
