# Supplementary material for: Time-Dependent, HIV-Tat-Induced Perturbation of Human Neurons In Vitro: Towards a Model for the Molecular Pathology of HIV-Associated Neurocognitive Disorders
Source: Front Mol Neurosci. 2017 May 29;10:163. doi: 10.3389/fnmol.2017.00163 (PMC5447036; doi:10.3389/fnmol.2017.00163)
Supplement: Supplementary file 2 [file Data_Sheet_2.docx]

Supplementary Material S-2

Time-dependent, HIV-Tat-induced perturbation of human neurons *in vitro*: a model for the molecular pathology of HIV associated neurocognitive disorders

Kim T. Gurwitz, Richard J. Burman, Brandon Murugan, Shaun Garnett, Tariq Ganief, Nelson C. Soares, Joseph V. Raimondo^*^, Jonathan M. Blackburn^*^

*** Correspondence:**

Jonathan M. Blackburn: jonathan.blackburn@uct.ac.za

Joseph V. Raimondo: joseph.raimondo@uct.ac.za

Content

| Mass spectrometry data quality.......................... | 2 |
| --- | --- |
| Biological significance analysis for 90% confluence time course……………………….. | 3 |
| Biological significance analysis for 70% time course………………………………………….. | 16 |


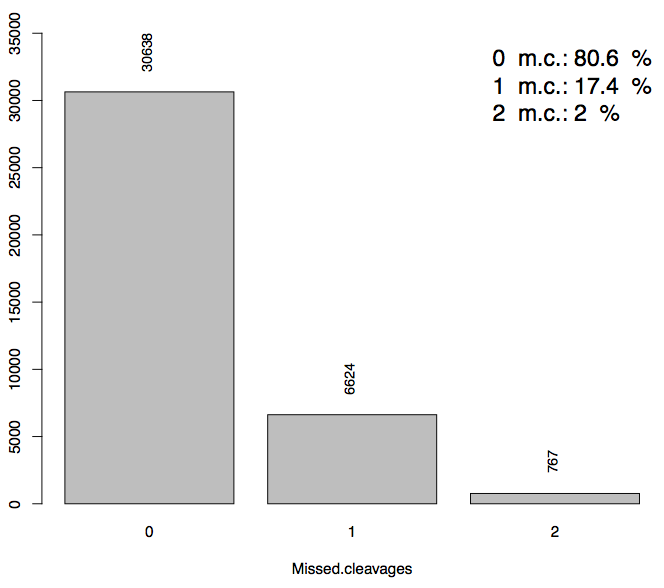


**Figure S-2.1. Tryptic digestion** m.c. missed cleavages


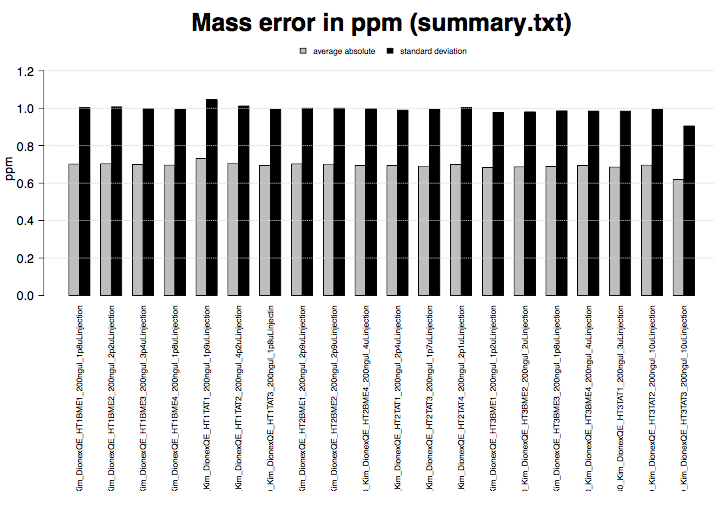


T1CON1

T1CON2

T1CON3

T1CON4

T1TAT1

T1TAT2

T1TAT3

T2CON1

T2CON2

T2CON4

T2TAT1

T2TAT3

T2TAT4

T3CON1

T3CON2

T3CON3

T3CON4

T3TAT1

T3TAT2

T3TAT3

**Figure S-2.2. Average absolute mass error of the MS instrument per run** ppm – parts per million, T1-3 – time point 1-3, TAT - HIV-Tat treated, CON - control.

**Table S-2.1** 6 hours post treatment**: Differentially expressed protein groups mapped to Panther protein class: *cytoskeletal protein* (PC00085)

| geneID | gene.name | p.value | log_2_FC* | subtype | PEP |
| --- | --- | --- | --- | --- | --- |
| MCA3 | Eukaryotic translation elongation factor 1 epsilon-1 | 0.016 | 0.18 | actin | 4.51e-28 |
| CTNA1 | Catenin alpha-1 | 0.037 | -0.23 | actin | 0 |
| DEST | Destrin | 0.0063 | 0.32 | actin | 1.58e-49 |
| LMNA | Prelamin-A/C | 0.030 | 0.20 | intermediary filament | 1.52e-44 |

*FC- fold change, HIV-Tat/ BME (i.e. treated/control)

** cells differentiated at 90% cell culture confluence

**Table S-2.2** 24 hours post treatment**: Differentially expressed protein groups mapped to

Panther protein class: *cytoskeletal protein* (PC00085)

| geneID | gene.name | p.value | log_2_FC* | subtype | PEP |
| --- | --- | --- | --- | --- | --- |
| ARP10 | Actin-related protein 10 | 0.023 | -0.079 | actin | 6.00e-129 |
| FMN2 | Formin-2 | 0.0047 | -0.46 | actin | 4.71e-18 |
| ZFHX3 | Zinc finger homeobox protein 3 | 0.032 | -0.11 | actin | 1.66e-13 |
| TWF1 | Twinfilin-1 | 0.026 | -0.11 | actin | 8.50e-141 |
| ADDA | Alpha-adducin | 0.032 | -0.16 | actin | 1.17e-121 |
| OPA1 | Dynamin-like 120 kDa protein, mitochondrial | 0.022 | -0.13 | microtubule | 8.28e-127 |
| GCP3 | Gamma-tubulin complex component 3 | 0.017 | -0.19 | microtubule | 3.79e-26 |
| KLC2 | Kinesin light chain 2 | 0.0053 | -0.18 | microtubule | 8.14e-106 |
| MAP1S | Microtubule-associated protein 1S | 0.033 | -0.18 | microtubule | 1.79e-115 |

*FC- fold change, HIV-Tat/ BME (i.e. treated/control)

** cells differentiated at 90% cell culture confluence

**Table S-2.3** 48 hours post treatment**: Differentially expressed protein groups

mapped to Panther protein class: *cytoskeletal protein* (PC00085)

| geneID | gene.name | p.value | log_2_FC* | subtype | PEP |
| --- | --- | --- | --- | --- | --- |
| CTNA1 | Catenin alpha-1 | 0.031 | -0.15 | actin | 0 |
| CTNA2 | Catenin alpha-2 | 0.038 | -0.19 | actin | 0 |

*FC- fold change, HIV-Tat/ BME (i.e. treated/control)

** cells differentiated at 90% cell culture confluence

**Table S-2.4** 6 hours post treatment**: Differentially expressed protein groups mapped to Panther protein class: *nucleic acid binding* (PC000171)

| geneID | gene.name | p.value | log_2_FC* | subtype | PEP |
| --- | --- | --- | --- | --- | --- |
| BAZ1B | Tyrosine-protein kinase BAZ1B | 0.021 | 0.16 | DNA | 9.10e-274 |
| PARP1 | Poly [ADP-ribose] polymerase 1 | 0.026 | 0.12 | DNA | 0 |
| MCM5 | DNA replication licensing factor MCM5 | 0.035 | 0.28 | DNA | 6.29e-161 |
| SMRC2 | SWI/SNF complex subunit SMARCC2 | 0.038 | 0.47 | DNA | 0 |
| PDS5B | Sister chromatid cohesion protein PDS5 homolog B | 0.0041 | 0.16 | DNA | 7.86e-164 |
| RFA1 | Replication protein A 70 kDa DNA-binding subunit | 0.040 | 0.14 | DNA | 2.79e-271 |
| BRD4 | Bromodomain-containing protein 4 | 0.038 | 0.28 | DNA | 2.36e-59 |
| RCC2 | Protein RCC2 | 0.043 | 0.16 | DNA | 0 |
| EDF1 | Endothelial differentiation-related factor 1 | 0.035 | 0.47 | other | 5.55e-30 |
| GMPPA | Mannose-1-phosphate guanyltransferase alpha | 0.022 | 0.15 | RNA | 2.68e-12 |
| MCES | mRNA cap guanine-N7 methyltransferase | 0.034 | 0.30 | RNA | 3.64e-64 |
| KHDR2 | KH domain-containing, RNA-binding, signal transduction-associated protein 2 | 0.041 | 0.56 | RNA | 2.53e-16 |
| PRP4 | U4/U6 small nuclear ribonucleoprotein Prp4 | 0.042 | 0.23 | RNA | 5.59e-67 |
| RBM12 | RNA-binding protein 12 | 0.014 | 0.27 | RNA | 1.02e-119 |
| MTA70 | N6-adenosine-methyltransferase 70 kDa subunit | 0.035 | 0.13 | RNA | 4.86e-42 |
| DX39A | ATP-dependent RNA helicase DDX39A | 0.0064 | 0.26 | RNA | 6.11e-210 |
| RL10A | 60S ribosomal protein L10a | 0.0147 | 0.11 | RNA | 1.16e-111 |
| MCA3 | Eukaryotic translation elongation factor 1 epsilon-1 | 0.016 | 0.18 | RNA | 4.51e-28 |
| THOC4 | THO complex subunit 4 | 0.028 | 0.35 | RNA | 1.23e-269 |
| SYK | Lysine--tRNA ligase | 0.013 | 0.13 | RNA | 6.43e-154 |
| RS2 | 40S ribosomal protein S2 | 0.018 | 0.14 | RNA | 8.09e-160 |
| EIF3L | Eukaryotic translation initiation factor 3 subunit L | 0.027 | 0.12 | RNA | 3.88e-244 |
| RL21 | 60S ribosomal protein L21 | 0.028 | 0.24 | RNA | 2.68e-71 |
| HNRH2 | Heterogeneous nuclear ribonucleoprotein H2 | 0.014 | 0.23 | RNA | 0 |
| EIF3C | Eukaryotic translation initiation factor 3 subunit C | 0.034 | 0.14 | RNA | 5.57e-146 |
| CPSF3 | Cleavage and polyadenylation specificity factor subunit 3 | 0.017 | 0.24 | RNA | 3.37e-101 |
| RL5 | 60S ribosomal protein L5 | 0.030 | 0.14 | RNA | 2.23e-174 |
| PSPC1 | Paraspeckle component 1 | 0.043 | 0.21 | RNA | 5.04e-258 |
| RBM8A | RNA-binding protein 8A | 0.022 | 0.31 | RNA | 2.03e-240 |
| HNRPK | Heterogeneous nuclear ribonucleoprotein K | 0.026 | -0.55 | RNA | 0 |
| DDX18 | ATP-dependent RNA helicase DDX18 | 0.044 | 0.22 | RNA | 8.05e-51 |

*FC- fold change, HIV-Tat/ BME (i.e. treated/control)

** cells differentiated at 90% cell culture confluence

**Table S-2.5** 24 hours post treatment**: Differentially expressed protein groups mapped to Panther protein class: *nucleic acid binding* (PC000171)

| geneID | gene.name | p.value | log_2_FC* | subtype | PEP |
| --- | --- | --- | --- | --- | --- |
| SMRD3 | SWI/SNF-related matrix-associated actin-dependent regulator of chromatin subfamily D member 3 | 0.017 | -0.20 | DNA | 1.04e-48 |
| TBPL1 | TATA box-binding protein-like protein 1 | 0.019 | 0.085 | DNA | 2.97e-58 |
| ZNF24 | Zinc finger protein 24 | 0.001 | 0.19 | DNA | 8.49e-05 |
| IF5 | Eukaryotic translation initiation factor 5 | 0.015 | -0.062 | RNA | 1.40e-130 |
| CHTOP | Chromatin target of PRMT1 protein | 0.0040 | -0.074 | RNA | 1.24e-91 |
| EIF3G | Eukaryotic translation initiation factor 3 subunit G | 0.032 | -0.085 | RNA | 4.00e-133 |
| RS16 | 40S ribosomal protein S16 | 0.018 | -0.11 | RNA | 2.23e-47 |
| ZFHX3 | Zinc finger homeobox protein 3 | 0.032 | -0.11 | RNA | 1.66e-13 |
| RU17 | U1 small nuclear ribonucleoprotein 70 kDa | 0.043 | -0.13 | RNA | 4.91e-130 |
| EFTU | Elongation factor Tu, mitochondrial | 0.032 | -0.13 | RNA | 0 |
| SYCC | Cysteine--tRNA ligase, cytoplasmic | 0.033 | -0.16 | RNA | 5.80e-85 |
| DDX18 | ATP-dependent RNA helicase DDX18 | 0.030 | -0.29 | RNA | 8.06e-51 |
| WDR7 | WD repeat-containing protein 7 | 0.043 | -0.29 | RNA | 1.43e-15 |
| RBM5 | RNA-binding protein 5 | 0.0016 | -0.32 | RNA | 4.14e-28 |
| LRRF1 | Leucine-rich repeat flightless-interacting protein 1 | 0.014 | 0.063 | RNA | 1.37e-13 |
| MRT4 | mRNA turnover protein 4 homolog | 0.028 | 0.26 | RNA | 1.51e-26 |
| EXOS8 | Exosome complex component RRP43 | 0.022 | 0.28 | RNA | 8.78e-75 |
| ERI3 | ERI1 exoribonuclease 3 | 0.001 | 0.45 | RNA | 2.85e-15 |

*FC- fold change, HIV-Tat/ BME (i.e. treated/control)

** cells differentiated at 90% cell culture confluence

**Table S-2.6** 48 hours post treatment**: Differentially expressed protein groups mapped to Panther protein class: *nucleic acid binding* (PC000171)

| geneID | gene.name | p.value | log_2_FC* | subtype | PEP |
| --- | --- | --- | --- | --- | --- |
| ATPB | ATP synthase subunit beta, mitochondrial | 0.028 | -0.046 | DNA | 0 |
| LN28B | Protein lin-28 homolog B | 0.0039 | 0.14 | DNA | 1.0045e-10 |
| HMGB2 | High mobility group protein B2 | 0.015 | 0.34 | DNA | 3.7012e-24 |
| SMRD1 | SWI/SNF-related matrix-associated actin-dependent regulator of chromatin subfamily D member 1 | 0.025 | 0.12 | DNA | 3.2527e-191 |
| MSH2 | DNA mismatch repair protein Msh2 | 0.036 | 0.21 | DNA | 2.0168e-159 |
| RUXF | Small nuclear ribonucleoprotein F | 0.043 | 0.53 | RNA | 2.1771e-80 |
| PRP16 | Pre-mRNA-splicing factor ATP-dependent RNA helicase PRP16 | 0.021 | 0.42 | RNA | 1.1609e-39 |
| SREK1 | Splicing regulatory glutamine/lysine-rich protein 1 | 0.042 | 0.1 | RNA | 1.8535e-49 |
| RL13 | 60S ribosomal protein L13 | 0.037 | 0.34 | RNA | 1.1597e-115 |
| CHTOP | Chromatin target of PRMT1 protein | 0.024 | 0.27 | RNA | 1.2434e-91 |
| IF2B1 | Insulin-like growth factor 2 mRNA-binding protein 1 | 0.042 | 0.27 | RNA | 0 |

*FC- fold change, HIV-Tat/ BME (i.e. treated/control)

** cells differentiated at 90% cell culture confluence

**Table S-2.7** Differentially expressed genes at 6 and 24 hours post HIV-Tat treatment***

| geneID | gene.name | Function* | log_2_FC** | p.value | PEP |
| --- | --- | --- | --- | --- | --- |
| **MRPL45** | 39S ribosomal protein L45, mitochondrial | Mitochondrial protein translation | HT1: 0.23 | HT1: 0.027 | 4.7e-07 |
|  |  |  | HT2: -0.2 | HT2: 0.0032 |  |
| **ACAD9** | Acyl-CoA dehydrogenase family member 9, mitochondrial | Catalyses rate limiting step in β oxidation | HT1: 0.087 | HT1: 0.013 | 2.9e-52 |
|  |  |  | HT2: -0.16 | HT2: 0.025 |  |
| **CSN3** | COP9 signalosome complex subunit 3 | Protein degradation | HT1: 0.15 | HT1: 0.039 | 5.6e-18 |
|  |  |  | HT2: 0.75 | HT2: 0.029 |  |
| **DDX18** | ATP-dependent RNA helicase | RNA helicase | HT1: 0.22 | HT1: 0.43 | 8.1e-51 |
|  |  |  | HT2: -0.28 | HT2: 0.029 |  |
| **GSTA4** | Glutathione S-transferase A4 | Detoxification of lipid peroxidation products | HT1: 0.16 | HT1: 0.011 | 3.0e-36 |
|  |  |  | HT2: 0.14 | HT2: 0.037 |  |
| **IGSF3** | Immunoglobulin superfamily member 3 | Cell surface receptor, immune system | HT1: -0.27 | HT1: 0.021 | 1.8e-34 |
|  |  |  | HT2: -0.38 | HT2: 0.0036 |  |
| **J3QRU4** | Vesicle-associated membrane protein 2 | Docking of vesicles with membrane at presynaptic vesicle for neurotransmitter release | HT1: -0.026 | HT1: 0.0080 | 9.9e-14 |
|  |  |  | HT2: -0.27 | HT2: 0.043 |  |
| **PABP1** | Polyadenylate-binding protein 1 | Translation initiation | HT1: 0.098 | HT1: 0.0036 | 0 |
|  |  |  | HT2: 0.052 | HT2: 0.020 |  |
| **VPS16** | Vacuolar protein sorting-associated protein 16 | Mediation of vesicle trafficking in endosome/ lysosome pathway (by homology) | HT1: 0.11 | HT1: 0.024 | 2.5e-18 |
|  |  |  | HT2: -0.24 | HT2: 0.029 |  |

* Function from GeneCards http://www.genecards.org/

**FC- fold change, HIV-Tat/ BME (i.e. treated/control)

***cells differentiated at 90% cell culture confluence

**Table S-2.8** Differentially expressed protein groups at both 24 and 48 hours post treatment***

| geneID | gene.name | Function* | log_2_FC** | p.value | PEP |
| --- | --- | --- | --- | --- | --- |
| **BRE1B** | E3 ubiquitin-protein ligase | ubiquitination and degradation of syntaxin 1 - essential component of the neurotransmitter release machinery  (by homology) | HT2: -0.16 | HT2: 0.015 | 2.43e-43 |
|  |  |  | HT3: 0.46 | HT3: 0.028 |  |
| **CHTOP** | Isoform 3 of Chromatin target of PRMT1 | Required for effective mRNA nuclear export | HT2: -0.074 | HT2: 0.0039 | 1.24e-91 |
|  |  |  | HT3: 0.27 | HT3: 0.023 |  |

* Function from GeneCards http://www.genecards.org/

**FC- fold change, HIV-Tat/ BME (i.e. treated/ control)

***cells differentiated at 90% cell culture confluence

**Table S-2.9** Differentially expressed genes at both 6 and 48 hours post treatment***

| geneID | Gene.name | Function* | p.value | Log_2_FC** | PEP |
| --- | --- | --- | --- | --- | --- |
| **CO3A1** | Collagen alpha-1(III) chain | Actin component | HT1: 0.032 | HT1: 0.58 | 1.3562e-33 |
|  |  |  | HT3: 0.029 | HT3: 0.44 |  |
| **CTNA1** | Catenin alpha-1 | Actin component | HT1: 0.037 | HT1: -0.23 | 0 |
|  |  |  | HT3: 0.030 | HT3: -0.15 |  |
| **SNX27** | Isoform 3 of Sorting nexin-27 | Endocytosis of plasma membrane receptors | HT1: 0.0057 | HT1: 0.49 | 9.8375e-18 |

* Function from GeneCards http://www.genecards.org/

**FC- fold change, HIV-Tat/ BME (i.e. treated/ control)

***cells differentiated at 90% cell culture confluence

**Table S-2.10** Top 10** output from over/ under representation analysis (i.e. Fisher exact test) of Panther protein class terms at 6 hours post treatment*

| PANTHER Protein Class | Background (out of 2104 protein groups) | Differentially expressed protein groups - input (out of 115 protein groups) | Differentially expressed protein groups - expected | over/  under representation | fold enrich-ment | p.value |
| --- | --- | --- | --- | --- | --- | --- |
| RNA methyltransferase (PC00033) | 2 | 2 | .11 | + | > 5 | 5.52E-03 |
| transcription cofactor (PC00217) | 24 | 5 | 1.31 | + | 3.81 | 1.05E-02 |
| hydrolase (PC00121) | 208 | 5 | 11.37 | - | .44 | 2.44E-02 |
| microtubule family cytoskeletal protein (PC00157) | 54 | 0 | 2.95 | - | < 0.2 | 5.03E-02 |
| cytoskeletal protein (PC00085) | 163 | 4 | 8.91 | - | .45 | 5.14E-02 |
| calcium-binding protein (PC00060) | 53 | 0 | 2.90 | - | < 0.2 | 5.32E-02 |
| RNA binding protein (PC00031) | 290 | 22 | 15.85 | + | 1.39 | 6.78E-02 |
| chromatin/chromatin-binding protein (PC00077) | 42 | 5 | 2.30 | + | 2.18 | 8.12E-02 |
| small GTPase (PC00208) | 30 | 4 | 1.64 | + | 2.44 | 8.30E-02 |
| nucleic acid binding (PC00171) | 447 | 31 | 24.43 | + | 1.27 | 8.59E-02 |

** please note that not all Panther protein classes listed are significantly over/ under represented (check p.value column)

*cells differentiated at 90% cell culture confluence

**Table S-2.11** Top 10** output from over/ under representation analysis (i.e. Fisher exact test) of Panther protein class terms at 24 hours post treatment*

| PANTHERProtein Class | Background (out of 2293 protein groups) | Differentially expressed protein groups - input (out of 97 protein groups) | Differentially expressed protein groups- expected | over/under representat-ion | fold enrich-ment | p.value |
| --- | --- | --- | --- | --- | --- | --- |
| DNA binding protein (PC00009) | 129 | 1 | 5.46 | - | < 0.2 | 2.47E-02 |
| esterase (PC00097) | 19 | 3 | .80 | + | 3.73 | 4.72E-02 |
| oxidoreductase (PC00176) | 147 | 2 | 6.22 | - | .32 | 4.77E-02 |
| exoribonuclease (PC00099) | 10 | 2 | .42 | + | 4.73 | 6.75E-02 |
| reductase (PC00198) | 58 | 0 | 2.45 | - | < 0.2 | 8.33E-02 |
| signaling molecule (PC00207) | 53 | 0 | 2.24 | - | < 0.2 | 1.03E-01 |
| hydrolase (PC00121) | 234 | 14 | 9.90 | + | 1.41 | 1.16E-01 |
| tyrosine protein kinase receptor (PC00233) | 3 | 1 | .13 | + | > 5 | 1.19E-01 |
| phosphorylase (PC00187) | 3 | 1 | .13 | + | > 5 | 1.19E-01 |
| Unclassified (UNCLASSIFIED) | 735 | 37 | 31.09 | + | 1.19 | 1.21E-01 |

** please note that not all Panther protein classes listed are significantly over/ under represented (check p.value column)

*cells differentiated at 90% cell culture confluence

**Table S-2.12** Top 10** output from over/ under representation analysis (i.e. Fisher exact test) of Panther protein class terms at 48 hours post treatment*

| PANTHER Protein Class | Background (out of 2022 protein groups) | Differentially expressed protein groups - input (out of 39 protein groups) | Differentially expressed protein groups - expected | over/under representation | fold enrichment | p.value |
| --- | --- | --- | --- | --- | --- | --- |
| cell adhesion molecule (PC00069) | 19 | 3 | .37 | + | > 5 | 5.89E-03 |
| extracellular matrix protein (PC00102) | 12 | 2 | .23 | + | > 5 | 2.26E-02 |
| extracellular matrix structural protein (PC00103) | 2 | 1 | .04 | + | > 5 | 3.79E-02 |
| peptide hormone (PC00179) | 2 | 1 | .04 | + | > 5 | 3.79E-02 |
| epimerase/racemase (PC00096) | 16 | 2 | .31 | + | > 5 | 3.82E-02 |
| antibacterial response protein (PC00051) | 3 | 1 | .06 | + | > 5 | 5.63E-02 |
| surfactant (PC00212) | 3 | 1 | .06 | + | > 5 | 5.63E-02 |
| DNA binding protein (PC00009) | 111 | 5 | 2.14 | + | 2.34 | 6.08E-02 |
| signaling molecule (PC00207) | 49 | 3 | .95 | + | 3.17 | 6.82E-02 |
| ligand-gated ion channel (PC00141) | 4 | 1 | .08 | + | > 5 | 7.43E-02 |

** please note that not all Panther protein classes listed are significantly over/ under represented (check p.value column)

* cells differentiated at 90% cell culture confluence

**Table S-2.13** Top 11** output from over/ under representation analysis (i.e. Fisher exact test) of Panther protein class terms at 6 hours post treatment*

| PANTHER Protein Class | Background (out of 1447 protein groups) | Differentially expressed protein groups - input (out of 31 protein groups) | Differentially expressed protein groups-expected | over/ under representat-ion | fold enrich-ment | p.value |
| --- | --- | --- | --- | --- | --- | --- |
| acyltransferase (PC00042) | 9 | 3 | .19 | + | > 5 | 9.49E-04 |
| ligand-gated ion channel (PC00141) | 4 | 2 | .09 | + | > 5 | 3.37E-03 |
| anion channel (PC00049) | 6 | 2 | .13 | + | > 5 | 7.38E-03 |
| ATP synthase (PC00002) | 7 | 2 | .15 | + | > 5 | 9.91E-03 |
| dehydrogenase (PC00092) | 48 | 4 | 1.03 | + | 3.89 | 1.86E-02 |
| ion channel (PC00133) | 11 | 2 | .24 | + | > 5 | 2.32E-02 |
| cation transporter (PC00068) | 12 | 2 | .26 | + | > 5 | 2.73E-02 |
| receptor (PC00197) | 32 | 3 | .69 | + | 4.38 | 3.07E-02 |
| histone (PC00118) | 13 | 2 | .28 | + | > 5 | 3.16E-02 |
| decarboxylase (PC00089) | 3 | 1 | .06 | + | > 5 | 6.23E-02 |
| DNA binding protein (PC00009) | 102 | 5 | 2.19 | + | 2.29 | 6.38E-02 |

** please note that not all Panther protein classes listed are significantly over/ under represented (check p.value column)

*cells differentiated at 70% cell culture confluence

**Table S-2.14** Top 10** output from over/ under representation analysis (i.e. Fisher exact test) of Panther protein class terms at 24 hours post treatment*

| PANTHER Protein Class | Background (out of 1945 protein groups) | Differentially expressed protein groups - input (out of 68 protein groups) | Differentially expressed protein groups- expected | over/ under represent-ation | fold enrichment | p.value |
| --- | --- | --- | --- | --- | --- | --- |
| extracellular matrix structural protein (PC00103) | 2 | 2 | .07 | + | > 5 | 2.30E-03 |
| centromere DNA-binding protein (PC00071) | 3 | 2 | .10 | + | > 5 | 5.07E-03 |
| surfactant (PC00212) | 3 | 2 | .10 | + | > 5 | 5.07E-03 |
| extracellular matrix protein (PC00102) | 12 | 3 | .42 | + | > 5 | 8.73E-03 |
| antibacterial response protein (PC00051) | 4 | 2 | .14 | + | > 5 | 8.81E-03 |
| glycosyltransferase (PC00111) | 17 | 3 | .59 | + | > 5 | 2.20E-02 |
| nuclear hormone receptor (PC00169) | 1 | 1 | .03 | + | > 5 | 3.44E-02 |
| ribonucleoprotein (PC00201) | 26 | 3 | .91 | + | 3.30 | 6.31E-02 |
| DNA ligase (PC00012) | 2 | 1 | .07 | + | > 5 | 6.76E-02 |
| receptor (PC00197) | 44 | 4 | 1.54 | + | 2.60 | 6.84E-02 |

** please note that not all Panther protein classes listed are significantly over/ under represented (check p.value column)

*cells differentiated at 70% cell culture confluence

**Table S-2.15** Top 10** output from over/ under representation analysis (i.e. Fisher exact test)

of Panther protein class terms at 48 hours post treatment*

| PANTHER Protein Class | Background (out of 1771 protein groups) | Differentially expressed protein groups - input (out of 89 protein groups) | Different-ially expressed protein groups - expected | over/ under represent-ation | fold enrich-ment | p.value |
| --- | --- | --- | --- | --- | --- | --- |
| nucleotide phosphatase (PC00173) | 5 | 3 | .25 | + | > 5 | 2.13E-03 |
| kinase activator (PC00138) | 9 | 3 | .45 | + | > 5 | 1.08E-02 |
| oxidoreductase (PC00176) | 111 | 1 | 5.58 | - | < 0.2 | 2.19E-02 |
| kinase modulator (PC00140) | 32 | 5 | 1.61 | + | 3.11 | 2.29E-02 |
| membrane traffic protein (PC00150) | 73 | 0 | 3.67 | - | < 0.2 | 2.36E-02 |
| defense/immunity protein (PC00090) | 13 | 3 | .65 | + | 4.59 | 2.82E-02 |
| chaperone (PC00072) | 61 | 7 | 3.07 | + | 2.28 | 3.42E-02 |
| dehydrogenase (PC00092) | 62 | 0 | 3.12 | - | < 0.2 | 4.19E-02 |
| deaminase (PC00088) | 7 | 2 | .35 | + | > 5 | 4.88E-02 |
| G-protein modulator (PC00022) | 42 | 5 | 2.11 | + | 2.37 | 6.09E-02 |

** please note that not all Panther protein classes listed are significantly over/

under represented (check p.value column)

*cells differentiated at 70% cell culture confluence

**Table S-2.16** 6 hours post treatment**: Differentially expressed protein groups mapped

to Panther protein class: *nucleic acid binding* (PC000171)

| geneID | gene.name | p.value | log_2_FC* | subtype | PEP |
| --- | --- | --- | --- | --- | --- |
| CHD7 | Chromodomain-helicase-DNA-binding protein 7 | 0.011 | 0.30 | DNA | 8.47e-24 |
| RAD21 | Double-strand-break repair protein rad21 homolog | 0.015 | -0.55 | DNA | 4.8e-95 |
| NXF1 | Nuclear RNA export factor 1 | 0.016 | -0.36 | RNA | 5.8e-54 |
| LSM2 | U6 snRNA-associated Sm-like protein LSm2 | 0.017 | 0.57 | RNA | 3.7e-44 |
| SUGP2 | SURP and G-patch domain-containing protein 2 | 0.022 | -0.40 | RNA | 2.3e-108 |
| H12 | Histone H1,2 | 0.027 | -0.41 | DNA | 0 |
| ROA2 | Heterogeneous nuclear ribonucleoproteins A2/B1 | 0.028 | -0.33 | RNA | 0 |
| VATB2 | V-type proton ATPase subunit B, brain isoform | 0.034 | -0.36 | DNA | 3.9e-149 |
| H2A1J | Histone H2A type 1-J | 0.034 | -1.25 | DNA | 7.4e-152 |
| ATPA | ATP synthase subunit alpha, mitochondrial | 0.045 | -0.36 | DNA | 0 |
| SRSF3 | Isoform 2 of Serine/arginine-rich splicing factor 3 | 0.047 | -0.42 | RNA | 1.2e-113 |

*FC- fold change, HIV-Tat/ control

**cells differentiated at 70% cell culture confluence

**Table S-2.17** 24 hours post treatment**: Differentially expressed protein groups

mapped to Panther protein class: *nucleic acid binding* (PC000171)

| geneID | gene.name | log_2_FC* | | p.value | | subtype | PEP |
| --- | --- | --- | --- | --- | --- | --- | --- |
| CBX8 | Chromobox protein homolog 8 | 0.26 | 0.0033 | | DNA | | 4.6e-10 |
| SRS10 | Isoform 5 of Serine/arginine-rich splicing factor 10 | 0.43 | 0.0049 | | RNA | | 2.3e-56 |
| DKC1 | H/ACA ribonucleoprotein complex subunit 4 | 0.12 | 0.0062 | | RNA/ DNA | | 1.7e-72 |
| COT1 | COUP transcription factor 1 | 0.44 | 0.0094 | | DNA | | 2.2e-88 |
| THOC4 | THO complex subunit 4 | 0.15 | 0.011 | | RNA | | 2.6e-227 |
| HNRPC | Isoform C1 of Heterogeneous nuclear ribonucleoproteins C1/C2 | 0.10 | 0.012 | | RNA | | 0 |
| RS17L | 40S ribosomal protein S17-like | 0.11 | 0.014 | | RNA | | 1.6e-42 |
| DHX9 | ATP-dependent RNA helicase A | 0.05 | 0.014 | | RNA | | 0 |
| TIAR | Nucleolysin TIAR | 0.44 | 0.021 | | RNA | | 1.4e-76 |
| MTA2 | Metastasis-associated protein MTA2 | 0.19 | 0.021 | | DNA | | 1.1e-84 |
| DNLI3 | DNA ligase 3 | 0.33 | 0.024 | | DNA | | 1.7e-61 |
| HNRPK | Isoform 3 of Heterogeneous nuclear ribonucleoprotein K | 0.31 | 0.028 | | DNA | | 0 |
| FUS | Isoform Short of RNA-binding protein FUS | 0.26 | 0.029 | | DNA | | 0 |
| BAZ1B | Isoform 2 of Tyrosine-protein kinase BAZ1B | 0.27 | 0.036 | | DNA | | 3.7e-104 |
| PRKRA | Interferon-inducible double-stranded RNA-dependent protein kinase activator A | 0.15 | 0.039 | | RNA | | 1.6e-33 |
| POGZ | Isoform 5 of Pogo transposable element with ZNF domain | 0.28 | 0.039 | | DNA | | 4.4e-83 |
| NOP58 | Nucleolar protein 58 | 0.23 | 0.042 | | RNA | | 2.5e-137 |
| YLPM1 | YLP motif-containing protein 1 | 0.23 | 0.045 | | DNA | | 4.5e-125 |

*FC- fold change, HIV-Tat/control

**cells differentiated at 70% cell culture confluence

**Table S-2.18** 48 hours post treatment**: Differentially expressed protein groups mapped to Panther protein class: *nucleic acid binding* (PC000171)

| geneID | gene.name | log_2_FC* | p.value | subtype | PEP |
| --- | --- | --- | --- | --- | --- |
| PRP31 | U4/U6 small nuclear ribonucleoprotein Prp31 | -0.29 | 0.0038 | RNA | 4.6e-34 |
| IF4E | Eukaryotic translation initiation factor 4E | -0.35 | 0.0039 | RNA | 3.5e-54 |
| LARP7 | La-related protein 7 | -0.22 | 0.0065 | DNA | 9.8e-65 |
| CPSF5 | Cleavage and polyadenylation specificity factor subunit 5 | -0.19 | 0.0072 | RNA | 6.4e-121 |
| IF1AX | Eukaryotic translation initiation factor 1A, X-chromosomal | -0.39 | 0.011 | RNA | 1.5e-39 |
| PSPC1 | Paraspeckle component 1 | -0.25 | 0.012 | DNA | 1.2e-151 |
| CRNL1 | Isoform 2 of Crooked neck-like protein 1 | -0.38 | 0.013 | RNA | 1.6e-32 |
| SMRCD | Isoform 3 of SWI/SNF-related matrix-associated actin-dependent regulator of chromatin subfamily A containing DEAD/H box 1 | -0.27 | 0.013 | DNA | 1.0e-25 |
| CPSF7 | Isoform 2 of Cleavage and polyadenylation specificity factor subunit 7 | -0.24 | 0.014 | RNA | 2.5e-94 |
| RL36L | 60S ribosomal protein L36a-like | -0.26 | 0.017 | RNA | 3.5e-31 |
| MTA2 | Metastasis-associated protein MTA2 | -0.14 | 0.018 | DNA | 1.1e-84 |
| C2AIL | CDKN2AIP N-terminal-like protein | 0.58 | 0.019 | DNA | 2.2e-24 |
| SYFB | Phenylalanine--tRNA ligase beta subunit | -0.22 | 0.019 | RNA | 2.8e-50 |
| ZCCHL | Zinc finger CCCH-type antiviral protein 1-like | -0.26 | 0.020 | DNA/ RNA | 1.8e-32 |
| ZN207 | Isoform 2 of BUB3-interacting and GLEBS motif-containing protein ZNF207 | -0.45 | 0.025 | DNA | 9.0e-74 |
| SMRD3 | Isoform 2 of SWI/SNF-related matrix-associated actin-dependent regulator of chromatin subfamily D member 3 | -0.23 | 0.026 | DNA | 2.7e-40 |
| IF4A3 | Eukaryotic initiation factor 4A-III | -0.19 | 0.026 | RNA | 2.1e-243 |
| PRKRA | Interferon-inducible double-stranded RNA-dependent protein kinase activator A | -0.40 | 0.027 | RNA | 1.6e-33 |
| CBX5 | Chromobox protein homolog 5 | -0.27 | 0.027 | DNA | 1.5e-95 |
| SF3B1 | Splicing factor 3B subunit 1 | -0.18 | 0.029 | RNA | 0 |
| DSRAD | Isoform 5 of Double-stranded RNA-specific adenosine deaminase | 0.29 | 0.032 | RNA | 1.5e-155 |
| UBP24 | Ubiquitin carboxyl-terminal hydrolase 24 | -0.25 | 0.035 |  | 1.2e-27 |
| EHMT1 | Histone-lysine N-methyltransferase EHMT1 | -0.16 | 0.036 | DNA | 2.0e-24 |
| CLIC4 | Chloride intracellular channel protein 4 | -0.16 | 0.039 |  | 1.4e-208 |
| ZFR | Zinc finger RNA-binding protein | -0.22 | 0.041 | RNA | 7.1e-128 |
| RL3 | 60S ribosomal protein L3 | -0.20 | 0.042 | RNA | 3.4e-131 |
| RBM25 | RNA-binding protein 25 | -0.26 | 0.044 | RNA | 6.0e-80 |

*FC- fold change, HIV-Tat/ control

**cells differentiated at 48 hours post treatment
